# Wnt/β-catenin pathway in arrhythmogenic cardiomyopathy

**DOI:** 10.18632/oncotarget.17457

**Published:** 2017-04-27

**Authors:** Alessandra Lorenzon, Martina Calore, Giulia Poloni, Leon J. De Windt, Paola Braghetta, Alessandra Rampazzo

**Affiliations:** ^1^ University of Padua, Department of Biology, Padua, Italy; ^2^ Maastricht University, Department of Cardiology, Maastricht, The Netherlands; ^3^ University of Padua, Department of Molecular Medicine, Padua, Italy

**Keywords:** Wnt, β-catenin, microRNAs, arrhythmogenic cardiomyopathy, molecular pathogenesis

## Abstract

Wnt/β-catenin signaling pathway plays essential roles in heart development as well as cardiac tissue homoeostasis in adults. Abnormal regulation of this signaling pathway is linked to a variety of cardiac disease conditions, including hypertrophy, fibrosis, arrhythmias, and infarction. Recent studies on genetically modified cellular and animal models document a crucial role of Wnt/β-catenin signaling in the molecular pathogenesis of arrhythmogenic cardiomyopathy (AC), an inherited disease of intercalated discs, typically characterized by ventricular arrhythmias and progressive substitution of the myocardium with fibrofatty tissue. In this review, we summarize the conflicting published data regarding the Wnt/β-catenin signaling contribution to AC pathogenesis and we report the identification of a new potential therapeutic molecule that prevents myocyte injury and cardiac dysfunction due to desmosome mutations *in vitro* and *in vivo* by interfering in this signaling pathway. Finally, we underline the potential function of microRNAs, epigenetic regulatory RNA factors reported to participate in several pathological responses in heart tissue and in the Wnt signaling network, as important modulators of Wnt/β-catenin signaling transduction in AC.

Elucidation of the precise regulatory mechanism of Wnt/β-catenin signaling in AC molecular pathogenesis could provide fundamental insights for new mechanism-based therapeutic strategy to delay the onset or progression of this cardiac disease.

## INTRODUCTION

The Wnt pathway is a highly conserved signaling pathway throughout evolution. It controls numerous stages of embryo development and adult tissue homeostasis through atiming- and location-specific activation [1, 2]. The pathway a timing is closely controlled by an overabundance of potential ligands, receptors, inhibitors, and second messengers acting as transcriptional regulators or post-translational modifiers. Thus, alterations of Wnt activity are often associated with developmental disorders and diseases, including cancer, neuronal diseases, and skeletal disorders [3–10]. Over the last decade, the role of Wnt signaling has been recognized also in cardiac pathophysiology and its dysregulation can result in processes leading to cardiac dysfunction, such as cardiac hypertrophy, fibrosis, arrhythmias, and infarction [11–13]. More recently, increasing evidence has indicated that the canonical Wnt/β-catenin pathway is implicated in the molecular pathogenesis of arrhythmogenic cardiomyopathy (AC) [14–15].

In this review, we aim to introduce the Wnt signaling pathway and to discuss its involvement in AC, together with the development of more selective therapeutic approaches based on Wnt targeting therapies.

### The Wnt signaling cascade

The Wnt pathway is coupled to a highly conserved family of secreted protein growth factors (cysteine-rich glycoproteins) known as Wnt proteins, that have been identified in animals from Hydra to Human [2, 16, 17]. The name Wnt, used in mammals, arises from the combination and the relationship between the *Drosophila melanogaster* segment polarity gene Wingless and its vertebrate orthologous, Int-1, a proto-oncogene discovered in 1984 in mouse [2, 18, 19]. At present, about 100 Wnt genes have been identified in various species, and 19 diverse Wnt proteins have been isolated in humans [16, 20–22]. Although Wnt family members show a high degree of sequence similarity, the expression of specific Wnt growth factors can lead to largely different signaling cascades [23].

At the cell membrane, Wnt ligands bind the Frizzled receptors (Fzd) as well as low-density Lipoprotein Receptor-related Protein-5 or -6 (LRP5/6) co-receptors. Fzd receptor family includes eleven members in Humans, which show seven transmembrane-spanning regions, an extracellular N-terminus Wnt-binding domain, and an intercellular C-terminal tail, crucial in the signal transduction [12, 24, 25].

Different Wnt ligands activate different intracellular signal transduction pathways, including the canonical β-catenin-dependent Wnt pathway, and the non-canonical β-catenin-independent Wnt/Planar cell polarity and Wnt-calcium/protein kinase C pathways [4, 26–28]. Wnt/Planar cell polarity (Wnt/PCP) and the Wnt/β-catenin pathway primarily regulates cell cycle-related proteins that are cell-type and context dependent [29], where as the Wnt/PCP is responsible for sensory cell orientation, cytoskeleton re-organization and directed migration [30]. The biological function of the Wnt/Calcium pathway, resulting in increased cytosolic Ca^2+^ concentration and activating Ca^2+^-sensitive proteins, is unclear, but it seems to be also involved in the controlling of cell fate and cell migration [26, 31].

In this review, we focus on the canonical Wnt pathway.

### Canonical Wnt pathway

**Figure 1:**
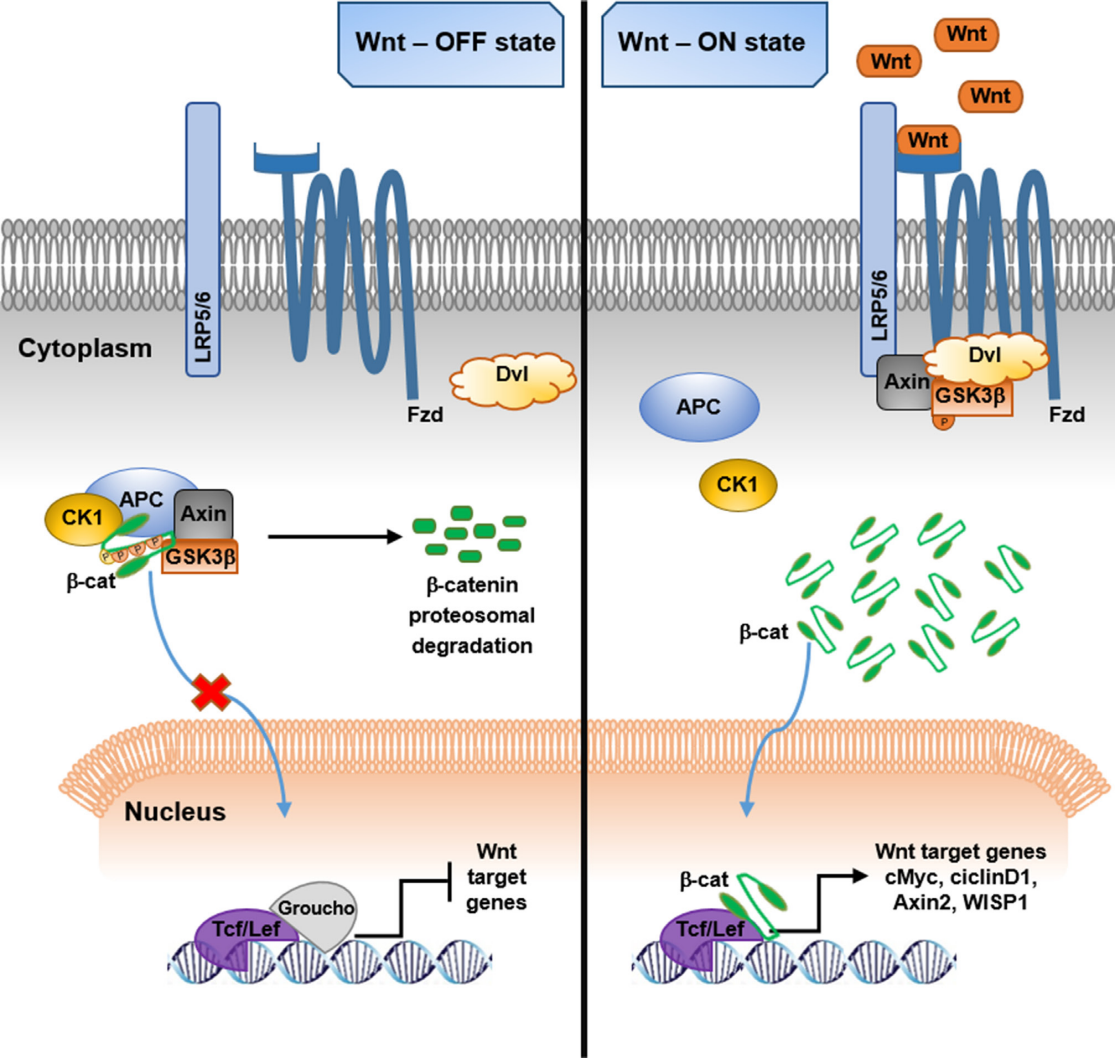
Schematic representation of β-catenin–mediated canonical Wnt pathway (Left) In the absence of Wnt ligands, the Wnt signaling is suppressed. Cytosolic β-catenin is phosphorylated by a destruction complex composed of Axin, APC, GSK3β and CK1 and then ubiquitinated and targeted to proteasomal degradation. Into the nucleus, the transcription of Wnt target genes is repressed by Groucho binding to Tcf/Lef. (Right) Wnt activation. The binding of Wnt ligands to their receptors Fzd/LRP5/6/Dvl, determines the disruption of the β-catenin destruction complex, thus inducing the stabilization of the protein, which can translocate into the nucleus, function as a cofactor for Tcf/Lef and activate Wnt target genes (APC, adenomatous polyposis coli; β-cat, β-catenin; CK1, casein kinase cell factor/lymphoid enhancer-binding factor).

The β-catenin protein is the main signal transducer in the canonical Wnt pathway (Figure 1). β-catenin is an Armadillo multifunctional protein that can play different roles in the cell, combining one as the crucial transcriptional activator mediating Wnt signal transduction, and another as a structural protein at cell-cell adhesion junctions [32].

In the canonical Wnt pathway, the absence of Wnt ligands determines the rapid phosphorylation of the cytosolic β-catenin protein by a cytoplasmic destruction complex. This complex consists of the scaffolding protein axin, adenomatous polyposis coli (APC) tumour suppressor protein, glycogen synthase kinase 3β (GSK3β), directly responsible for β-catenin phosphorylation at key amino-terminal Ser33, Ser37 and Thr41 residues, and casein kinase 1 (CK1), which phosphorylates β-catenin at the amino acid Ser45 [22, 33]. The post-translational phosphorylation of the protein leads to its ubiquitination and subsequent proteosomal degradation thus preventing its translocation in the nucleus, where, as a consequence, the binding of Groucho to T cell factor/lymphoid enhancer-binding factor (Tcf/Lef) blocks the transcription of Wnt responsive genes [34, 35] (Figure 1).

When Wnt ligands bind to Fzd receptor and LRP5/6 co-receptors, the Wnt signaling is activated. The binding of Wnt recruits the cytoplasmic protein dishevelled (Dvl) to the cell membrane, thus sequestering the rate-limiting components axin and GSK3β from the degradation complex and determining the destruction of the complex [36, 37]. The hypo-phosphorylated/stabilized form of cytosolic β-catenin accumulates and translocates to the nucleus, where Wnt responsive genes transcription is activated [22, 34] (Figure 1). Multiple target genes include WNT1-inducible signaling-pathway protein 1 (WISP1), cell cycle-related proteins (c-Myc, cyclin D1) and the negative regulator Axin2 [12].

### Wnt/β-catenin in the heart

#### Wnt signaling in heart development

Heart development and morphogenesis are meticulously controlled by the relationship of several signaling pathways that cooperate to provide cell fate specification. The rigorous expression of Wnt growth factors, together with several families of secreted factors including TGFbs (Transforming Growth Factor-Betas), FGFs (Fibroblast Growth Factors), BMPs (bone morphogenetic proteins), Notch and Hippo proteins, is essentially responsible for heart processes, including myocardial specification and proliferation, cardiac septation, chamber and cardiac valve formation, as well as endothelial and vascular smooth muscle cell proliferation [1, 11, 38–40].

Knocking out β-catenin results in mouse embryonic defects at gastrulation, not allowing the investigation of a possible Wnt function during cardiogenesis [41]. Additional developmental studies using inducible promoters to activate or repress Wnt/β-catenin signaling in zebrafish embryos at different times of development and studies in mouse and human embryonic stem cells demonstrate opposite and temporally distinct effects for Wnt/β-catenin signaling during early cardiogenesis. Wnt/β-catenin signaling at pregastrula stages appears to be required for subsequent heart precursor specification, whereas this pathway inhibits heart formation during gastrulation. This suggests that cells destined to become cardiomyocytes are redirected by Wnt signaling towards an alternate mesoderm fate [42–44]. On the other hand, premature suppression of the Wnt signaling prevents the proper formation of endocardial cushions and heart valves, and can also result in malformation of the outflow tract, including lack of septation of the large arterial trunks, transposition of the great arteries or double outlet right ventricle [45, 46]. An upregulation of specific Wnt ligands has been observed during differentiation of embryonic cardiomyocytes into Purkinje fibres, suggesting a regulative role of the Wnt signaling in the proper development of the cardiac conduction system [11].

A delicate balance between canonical and noncanonical Wnt pathways has been implicated in cardiac precursor differentiation, as well as in epicardium formation and differentiation. The canonical Wnt signaling appears to retain the cardiac precursors in a proliferative and precursor state [43], whereas the noncanonical signaling promotes their differentiation [47, 48]. In the epicardium organization, the Wnt/β-catenin signaling is essential in pro-epicardial and epicardial cell expansion and Wnt/PCP signaling in epicardial-derived cell differentiation [49].

The Wnt/β-catenin signaling also controls cardiomyocyte proliferation [50]. *In vitro* treatment of neonatal and adult rat cardiomyocytes with BIO, a pharmacological agent inhibiting GSK3, results in increased β-catenin activity and promotes cell proliferation and mitosis. Thus, the activation of canonical Wnt signaling participates in controlling the proliferative capability of the differentiated cardiomyocytes by promoting maintenance of stem cell properties [51]. In embryonic mouse hearts, the inactivation of Hippo, a cardiomyocyte growth and organ-size control pathway, leads to overgrown hearts due to increased stimulation of cardiomyocyte proliferation. In such hearts β-catenin and Wnt target genes are overexpressed, indicating that Wnt/β-catenin signaling might regulate pro-growth genes transcription, under an accurate control of Hippo pathway [52].

Wnt/β-catenin reporter mice unveiled the spatial and temporal distribution of the endogenous status of β-catenin activation in several tissues. Transgenic animals exhibit an evident reporter gene activity in the cardiac region, where it persists up to E10.5, whereas the Wnt/β-catenin pathway appears not active in the adult heart [53]. Therefore, while the key role of Wnt/β-catenin signaling in several processes of early stages of cardiac development has been well documented, its activation in the adult heart is mainly associated with pathological conditions.

### Wnt/β-catenin signaling in cardiac disease and repair

The adult mammalian heart includes interacting populations of cells, predominantly myocytes, fibroblasts, smooth muscle cells, endothelial and epicardial cells, all arranged in a precise three-dimensional network. Under pathological conditions, they can interact with each other directly or indirectly, through the expression of growth factors and cytokines, to affect a physiological cardiac response.

In terminally differentiated cardiomyocytes, the canonical Wnt signaling is normally quiescent [53], but it becomes reactivated upon pathological stress, including hypertrophy. Cardiac hypertrophy is characterized by an increase in cell size and upregulation of fetal-gene expression [54]. It can arise from physiological myocyte growth or, on the other hand, from pathological cardiac remodelling induced by cardiovascular diseases, often resulting in fibrosis substitution, diastolic dysfunction, and, eventually, heart failure [55–57]. Suppression of the signaling by overexpressing GSK3β, the key molecule regulating canonical Wnt transduction, attenuates the stress-induced hypertrophic response of ventricular cardiomyocytes in transgenic mice and leads to the development of smaller heart with markedly depressed contractility [58]. Both myocyte-specific deletion of β-catenin and lack of the Dvl-1 protein gene, responsible for the interruption of the Wnt pathway, also result in a significantly decreased hypertrophic response to pressure overload in transgenic mice, when compared with *wild-type* littermates [59, 60]. On the contrary, inhibition of GSK3β or overexpression of Dvl-1leads to augmented hypertrophy, severe systolic and diastolic dysfunction and progressive heart failure, in a pressure overload rat model and in transgenic mice, respectively [56, 61].

Many studies have emphasized an important role for the canonical Wnt signaling in cardiac fibrogenesis [49, 62, 63]. Fibrosis deposition can result from both cardiac repair post myocardial infarction and cardiac diseases, and it generally impedes electrical wave propagation, potentially causing arrhythmias [64, 65]. The aberrant activation of Wnt signaling by overexpression of Wnt ligands or nuclear accumulation of β-catenin in different experimental models, suggests that the canonical Wnt signaling alone is sufficient to promote the expression of a fibrogenic program in fibroblasts of several tissues [66, 67]. In epicardial cells of post myocardial infarcted heart, Wnt signaling is enhanced and promotes epithelial–mesenchymal transition into fibroblasts [49]. Accordingly, in paediatric heart allographs with diastolic dysfunction and severe epicardial fibrosis involving either epicardial surface or underlying adipose tissue or both, immunohistochemical investigation revealed nuclear accumulation of β-catenin and TCF4 (T-cell factor 4, the main Wnt pathway effector) in fibroblasts, consistent with the activation of Wnt signaling in these cells [63].

Moreover, Wnt signaling has been suggested to inhibit the apoptotic process involved in the transition from hypertrophy to heart failure. Indeed, in heart from patients who have developed heart failure, Akt activation and consequent GSK3β inhibition seem to protect cardiac cells from apoptosis [68].

Wnt1, a major element of the early Wnt/β-catenin signaling, is a specific and potent inducer of connexin-43 expression in cardiomyocytes. This effect results in enhanced accumulation of connexin-43 protein and formation of functional gap junction channels, supporting the hypothesis that dysregulated signaling contributes to altered impulse propagation and arrhythmia in the myopathic heart [69].

In conclusion, the Wnt/β-catenin signaling is a very complex system involved in a variety of cardiac pathologies with quite varied effects, according with the cell system and timing of intervention. For this reason, it can offer possibilities for new insights into disease pathogenesis and identification of new therapeutic targets.

### Arrhythmogenic cardiomyopathy

Arrhythmogenic cardiomyopathy (AC) is a rare, inherited cardiac disease characterized by ventricular arrhythmias, right ventricular dysfunction and sudden cardiac death [70, 71]. It has a prevalence between 1:2000 and 1:5000 and accounts for up to 10% of deaths from undiagnosed cardiac disease in the < 65 age group [72, 73]. Clinical diagnosis of AC is often difficult because of the non-specific nature of the disease features and the broad spectrum of phenotypic expressions, ranging from concealed to severe forms.

The early “concealed” phase is characterized by propensity toward ventricular tachyarrhythmia in the setting of well-preserved structural morphology and ventricular function. As the disease progresses, however, myocyte loss, inflammation, and accumulation of fibrofatty scar tissue become evident [74]. The typical pathological profile consists of the progressive replacement of the myocardium by fatty or fibrofatty tissue, starting from the epicardium or midmyocardium and then extending to became transmural. The infiltration may be diffuse or regional and is located at the angles of the so-called “triangle of dysplasia”, including the inferior, apical and infundibular walls [75]. Recent observations in genotyped AC patients revealed a characteristic pattern of cardiac disease that involves the epicardial surface of the basal right ventricle and lateral left ventricle, displacing the right ventricular apex [76].

A scoring system to establish the diagnosis of AC has been developed on the basis of the fulfilment of major and minor criteria encompassing structural, histological, electrocardiographic, arrhythmic and genetic features of the disease [77, 78].

**Figure 2:**
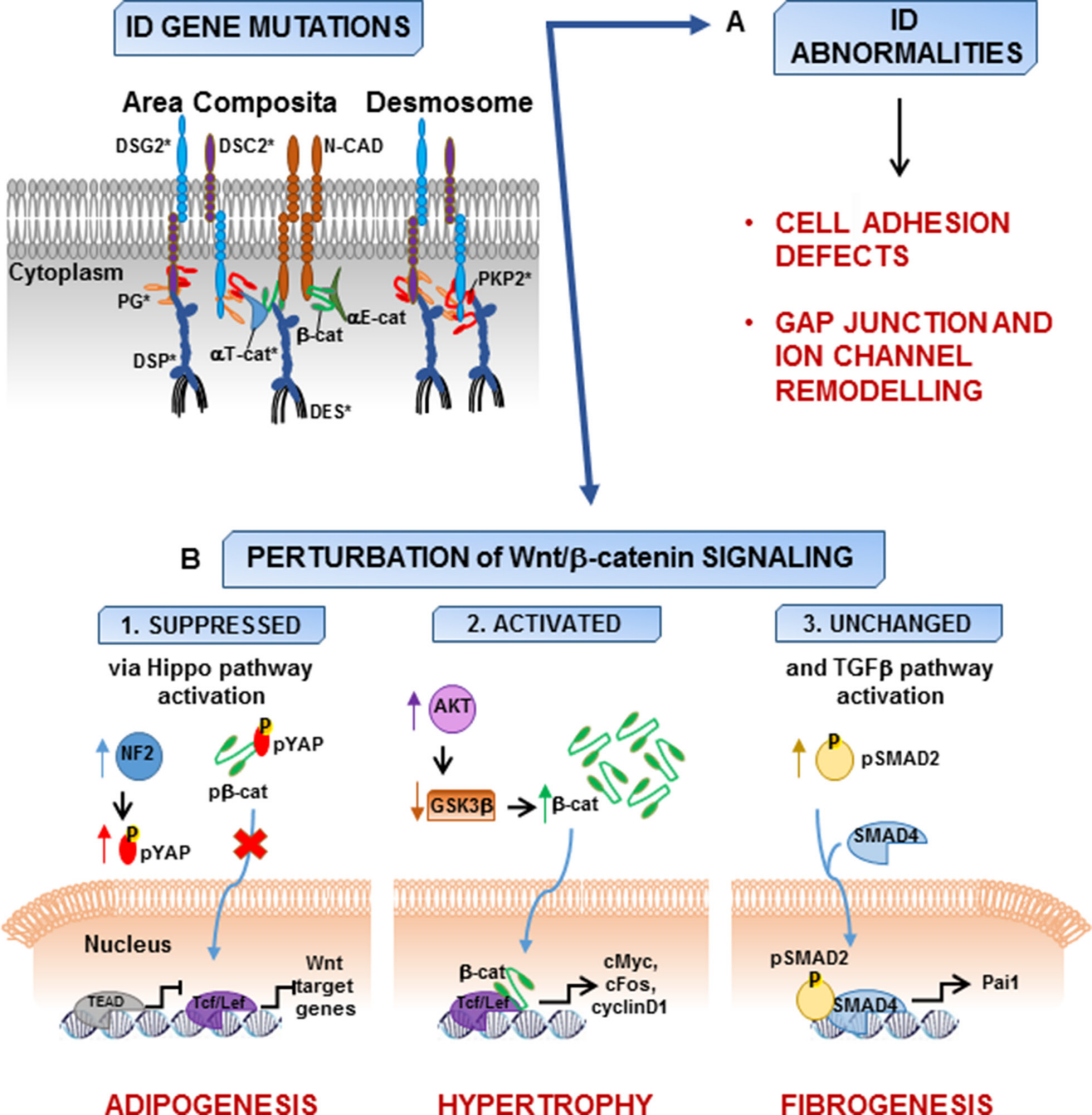
Models of AC pathogenesis AC gene mutations involve *area composita* and desmosome proteins (asterisks) and lead to abnormalities in intercalated disc (ID) consisting of cell adhesion defects, gap junction and ion channel remodelling. (**A**) Mutations in ID proteins can result also in the perturbation of the Wnt/β-catenin signaling. (**B**) Wnt/β-catenin signaling is suppressed in concomitance with the activation of Hippo pathway [93]. The impaired localization of phosphorylated protein kinase C-α to the perturbed IDs is associated with activation of neurofibromin 2 (NF2) and results in cascade phosphorylation leading to increased phospho-Yes-associated protein (pYAP) and its retention in the cytoplasm. pYAP interacts with β-catenin (β-cat), which is not able to translocate into the nucleus and, as a consequence, the expression of the effectors of both Hippo and canonical Wnt pathways (TEAD and Tcf/Lef, respectively) is suppressed and adipogenesis is enhanced (B1). Other experimental data reveal that loss of plakoglobin leads to the activation of AKT and the subsequent inhibition of glycogen synthase kinase 3β (GSK3β) resulting in the stabilization of β-cat and its translocation in the nucleus. Here, β-cat interacts with Tcf/Lef causing the increase of the expression of c-myc, c-fos, and cyclin D1, as well as cardiac hypertrophy [98] (B2). The disruption of junction integrity can result in increased presence of β-catenin at IDs without the involvement of Wnt/β-catenin signaling. However, increased expression of transforming growth factor β-1 (TGFβ1), phospho-SMAD2 (pSMAD2), and Pai1 is consistent with the activation of TGFβ pathway responsible for the progressive fibrosis in AC hearts [99] (B3) (DSC2, desmocollin-2; DSG2, desmoglein-2; DSP, desmoplakin; PKP2, plakophilin-2; DES, desmin; N-cad, N-cadherin; αT-cat, αT-catenin; αE-cat, αE-catenin; Tcf/Lef, T cell factor/lymphoid enhancer-binding factor; TEAD, SV40 transcriptional enhancer factor domain).

AC is an inherited cardiomyopathy characterized by incomplete penetrance and variable expressivity and it is usually transmitted as an autosomal dominant trait, even though recessive forms have been reported [15]. Since 1994, when the first locus was mapped [79], 13 disease genes have been identified [80] (Table 1). One or more disease-causing mutations are detected in about 60% of the patients [81] and most of them affect genes encoding for mechanical junction proteins, including desmoplakin (DSP) [82], plakophilin-2 (PKP2) [83], desmoglein-2 (DSG2) [84], desmocollin-2 (DSC2) [85], plakoglobin (JUP) [86] and alpha-T catenin (CTNNA3) [87]. In cardiomyocytes, mechanical and electrochemical coupling between adjacent cells relies on complex entities called intercalated discs (IDs), which in turn comprise different kinds of junctional apparatuses, namely desmosomes, gap junctions and the recently described *area composita* (this latter found only in cardiac muscle) [88] (Figure 2). The main components of cardiac desmosomes are two types of cadherins, desmocollin-2 and desmoglein-2, two armadillo proteins, plakoglobin and plakophilin-2, and desmoplakin. Function-wise, desmosomal cadherins are responsible for the cell-cell adhesion *via* their extracellular domains, while their cytoplasmic domains interact with the armadillo proteins. These latter interact with desmoplakin, which in turn binds desmin intermediate filaments, thereby creating a continuous link between the cytoskeletons of adjacent cardiomyocytes. The *area composita* comprises some of the desmosomal proteins described above as well as proteins normally found in *adherens* junctions. Amongst these, N-cadherin interacts with the adjacent cell *via* its extracellular domain and, on the cytoplasmic side, is indirectly linked to α-catenin *via* β-catenin or plakoglobin. In turn, α-catenin binds the F-actin cytoskeleton through vinculin [88].

**Table 1:** Human genes associated with AC

GENE		CHOMOSOME LOCUS	ENCODED PROTEIN	MUTATION FREQUENCE	REFERENCE(S)
INTERCALATED DISC GENES	**DSP**	6q24	Desmoplakin	1–16%	[82, 84, 117–119]
	**PKP2**	12p11	Plakophilin-2	10–45%	[83, 118–121]
	**DSG2**	18q12	Desmoglein-2	7–12%	[84, 118, 122]
	**DSC2**	18q12	Desmocollin-2	1–5%	[85, 121–123]
	**JUP**	17q21	Plakoglobin	1%	[86, 118]
	**TMEM43**	3p25	Luma	rare	[124]
	**DES**	2q35	Desmin	rare	[125, 126]
	**CTNNA3**	10q21	αT-catenin	2%	[87]
OTHER GENES	**RYR2**	1q42-q43	Cardiac ryanodine receptor	rare	[127]
	**TGFb3**	14q23-q24	Transcription growth factor β-3	rare	[128]
	**TTN**	2q31	Titin	rare	[129]
	**LMNA**	1q21.2-q21.3	Lamin A/C	rare	[130]
	**PLN**	6q22.1	Phospholamban	rare	[131]

As mentioned above, AC is often caused by mutations in genes involved in cell-cell adhesion. Indeed, a recent report has demonstrated that siRNA-mediated silencing of the PKP2 gene in neonatal rat ventricular myocytes subjected to mechanical stress led to a much greater destabilization of the monolayer structure, thereby indicating weakened cell-cell junctions [89]. However, other recent evidences suggest that the pathogenetic role of ID genes' mutations in AC does not originate exclusively from the mechanical alteration of the junctions but also from alterations in the signal transduction processes. For example, mutations in several of the ID genes have been associated to perturbation of the Wnt/β-catenin signaling (see below for specific references).

### Wnt signaling in AC: conflicting evidences

In the last years, studies on zebrafish and mouse models and on cultured cell lines, such as induced pluripotent stem cells (iPSCs) from affected patients, revealed the involvement of the canonical Wnt signaling in the molecular pathogenesis of AC (Table 2).

**Table 2:** Studies of the Wnt/β-catenin signaling involvement in AC

HUMAN AND ANIMAL EXPERIMENTAL MODEL(S)	SIGNALING COMPONENTS	PHYSIOLOGICAL EFFECT	CONCLUSIONS	REFERENCE(S)
DSP-deficient HL-1 cells Tg mice with cardiac-restricted DSP deletion (DSP^−/−^; DSP^+/−^)	Wnt: ↓cyclinD1, c-Myc Adipogenesis: ↑PPARγ, CEBPα, adiponectin	Fat droplet accumulation in DSP-deficient cells High lethality in DSP^−/−^ embryos Ventricular dilatation and disfunction, fibrofatty replacement of myocytes in DSP^−/−^and DSP^+/−^ adult mice	Nuclear PG translocation **Suppression of canonical Wnt/β-catenin signaling** Transcriptional switch to adipogenesis and fibrogenesis	[14]
Tg mice overexpressing cardiac truncated PG	In cardiac progenitor cells Wnt: ↓CTGF Adipogenesis: ↑KLF15, IGFBP5	Increased fibroadiposis, cardiac dysfunction, and premature death Enhanced adipogenesis in cardiac progenitor cells	**Suppression of canonical Wnt signaling** Induction of pro-adipogenic gene expression	[92]
Conditional Tg mice with cardiac-restricted JUP deletion	Wnt: ↑active β-catenin, c-Myc, c-Fos; ↓GSK3β, ↑AKT	Progressive loss of cardiac myocytes, extensive inflammatory infiltration, fibrous tissue replacement, and cardiac dysfunction	**Activation of canonical Wnt signaling**	[98]
Tg mice with cardiac-restricted JUP deletion	Wnt: ↑β-catenin, but unchanged cyclinD1, c-Myc TGFβ: ↑p-Smad2, TGFβ1, BNP, ANP	Cardiac fibrosis and dysfunction, ventricular arrhythmias Absence of desmosomes to the intercalated discs of cardiomyocytes	**Wnt signaling not altered** Increase of TGFβ signaling	[99]
iPS-CMs from patient carryingthe heterozygous PKP2 p.K672Rfs*12mutation; iPS-CMs from patient carryingthe homozygous PKP2 p.G828Gmutation	Wnt: ↓β-catenin Upon lipogenic stimulation: Adipogenesis: ↑PPARα, PPARγ, FABP4	Intracellular lipid accumulation Accelerated pathogenesis upon lipogenic stimulation	Nuclear PG translocation **Reduction of Wnt signaling** Induction of lipogenesis and apoptosis	[102, 104]
iPS-CMs from patient carryingthe homozygous PKP2 p.A324fs335*mutation; iPS-CMs from patient carrying the homozygous PKP2 p.T50Sfs60* mutation	Wnt: ↓β-catenin Adipogenesis: ↑PPARγ	Intracellular lipid accumulation Desmosomal distortion Accelerated pathogenesis upon adipogenic stimulation Reversion of intracellular lipid accumulation upon treatment with a GSK3β inhibitor (BIO)	**Reduction of Wnt signaling** Pro-adipogenic potential Activation of Wnt pathway rescues the lipid accumulation	[103]
Tg mice with cardiac restricted DSP deletion (DSP^+/−^)[14]; Tg mice overexpressing cardiac truncated PG [92]; PKP2 knockdown HL-1 cells AC patient myocardial samples	Hippo: ↓pPKC-α, ↑NF2, ↑pYAP Wnt: ↑pβ-catenin	Molecular remodeling of IDs No discernible localization of pPKC-α to IDs membrane localization of pYAP Cytoplasmatic binding of pYAP, pβ-catenin and JUP	Activation of Hippo pathway **Suppression of canonical Wnt signaling** Enhanced adipogenesis	[93]
myocardial samples from Boxer dogs affected with AC	Wnt: ↑β-catenin	β-catenin retention to the endoplasmic reticulum of cardiomyocytes	**Perturbation of canonical Wnt signaling**	[100]
Tg zebrafish expressing cardiac truncated PG (p.W680Gfs*11 mutation) Neonatal rat ventricular myocytes expressing cardiac truncated PG (p.W680Gfs*11 mutation) iPS-CMs from patient carryingthe heterozygous PKP2 p.K672Rfs*12 mutation iPS-CMs from patient carryingthe heterozygous PKP2 p.Q617* mutation AC patient myocardial samples	/	Reduction in I_Na_and I_K1_ current densities, interruptions in cell boundaries, structural disarray in fishes and in neonatal rat cardiomyocytes; Abnormal subcellular distribution of PG, connexin-43, Nav1.5 and SAP97 in cultured cells lines Reversion of AC features upon treatment with SB216763 (a GSK3β inhibitor)	Activation of Wnt pathway (by SB216763) rescues the AC pathobiological features	[96]
Tg mice expressing truncated Dsg2 Tg mice expressing cardiac truncated PG (JUP^2157del2^) AC patient myocardial samples Neonatal rat ventricular myocytes expressing cardiac truncated PG or PKP2 PKP2-knockdown HL-1 cells	Wnt: ↑GSK3β Upon SB216763 treatment: Wnt: ↓GSK3β, but unchanged pGSK3β	GSK3β ID redistribution in AC cardiomyocytes and in AC human myocardium, but not in normal cardiomyocytes	Activation of Wnt pathway by pharmacologic GSK3β inhibition (using SB216763) improves cardiomyopathy	[97]
Conditional Tg mice with heterozygous DSP deletion in fibroadipocyte progenitor cells (FAPs)	In FAPs: Wnt: ↓cyclinD1, CTGF, Serpine1 Adipogenesis: ↑FABP4, CEBPα, PPARγ	Mild cardiac dysfunction and increased cardiac fibroadipogenesis	**Suppression of canonical Wnt signaling** in FAPs Enhanced adipogenesis	[94]
Tg mice overexpressing cardiac mutant DSP^R2834H^ exposed to endurance exercise	Wnt: ↓β-catenin, pGSK3β, pAKT1	Progression of AC phenotype (right ventricle dilatation and wall thinning, myocyte disarray and fibrofatty infiltration)	Exercise-induced **suppression of the Wnt signaling**	[95]

The progressive replacement of the cardiomyocytes by adipocytes and fibrosis is the pathological hallmark of AC, which contributes to both cardiac dysfunction and arrhythmias. In this regard, in addition to the inactivation of junctional mechanical functions, which is supposed to lead to myocyte death under physical stress, the alteration of canonical Wnt/β-catenin signaling could act as a major transcriptional switch regulator of myogenesis towards adipogenesis [90].

Several studies from Marian's group demonstrated the suppression of Wnt/β-catenin/Tcf/Lef pathway in AC due to nuclear translocation of plakoglobin [14, 91, 92]. Plakoglobin, also known as γ-catenin, is highly homologous to β-catenin, the effector of the canonical Wnt signaling, and shares with β-catenin common protein partners thus fulfilling some of the same functions [32]. Suppression of desmoplakin expression in cultured atrial myocytes and in mouse hearts causes the impairment of junction assembly and frees plakoglobin from the cell membrane. Consequently, plakoglobin translocates into the nucleus where it interferes with β-catenin/Tcf transcriptional activity leading to an adipogenic switch and explaining enhanced adipogenesis, fibrogenesis, and myocyte apoptosis observed in human AC [14]. Thereafter, through a series of genetic-fate mapping experiments, the same group indicated that cardiac progenitor cells of the embryonic second heart field represent a cell source for excess adipocytes observed in the heart of Dsp^+/−^ mouse model for AC [91]. Direct evidence for the essential function of nuclear plakoglobin in repressing Wnt signaling in cardiac progenitor cells and inducing differentiation to adipocytes was demonstrated in hearts from transgenic mice overexpressing cardiac truncated plakoglobin [92]. Furthermore, the activation of Hippo pathway, another regulator of cellular differentiation and proliferation, was identified as the mechanism for suppression of the canonical Wnt signaling in AC. Pathogenic activation of the Hippo kinase cascade, resulting from perturbations in ID attributable to AC gene mutations, leads to phosphorylation and cytoplasmic retention of the transcriptional coactivator Yes-associated protein (YAP), that can sequester β-catenin and plakoglobin, thus resulting in a reduction of both β-catenin/TCF and YAP/TEAD transcriptional activities and enhanced heart adipogenesis [93] (Figure 2).

Very recently, a conditional mouse with cardiac deletion of Dsp was crossed to the PDRFRA (platelet-derived growth factor receptor-α)-Cre deleter mice in order to obtain a novel mouse model with traceable cardiac fibroadipocyte progenitors (FAPs) lacking of Dsp. These mice showed increased fibroadipogenesis in the heart and mild cardiac dysfunction, if compared with control mice. FAPs isolated from the same mouse hearts showed enhanced differentiation to adipocytes through a Wnt-dependent mechanism [94].

Finally, Wnt pathway perturbation, due to reduced AKT and GSK3β activation, has been recently associated to exercise-induced acceleration of AC phenotype in mice overexpressing a mutant form of desmoplakin and exposed to a daily running [95]. The identification of a Wnt pathway activator as the molecule that can rescue the features of the AC phenotype both in a zebrafish and in additional mouse models [96, 97] strongly supports the association between AC and suppression of this signaling pathway.

On the other hand, conflicting evidences have been reported about the canonical Wnt signaling involvement in AC pathogenesis. In a conditional mouse model with cardiac-restricted JUP deletion showing cardiac alterations and dysfunctions similar to those of AC patients, the remodeling of the IDs causes an increased β-catenin stabilization associated with activated AKT and consequent inhibition of GSK3β. In these animals, a Wnt/β-catenin signaling activation was suggested to contribute to the AC cardiac phenotype [98] (Figure 2). On the contrary, in a different mouse model with stable cardiac-restricted JUP deletion, the increase of β-catenin levels at IDs of cardiomyocytes does not affect the canonical Wnt signaling; the TGFβ pathway, mainly involved in the regulation of myocyte cell death, including both apoptosis and necrosis, and influencing cardiac fibrosis and hypertrophy, results upregulated during the early stages of the disease in mutant hearts [99] (Figure 2). A 50% increase in levels of β-catenin protein, that resulted mislocalized to the endoplasmic reticulum, is also observed in heart samples from Boxer dogs affected with AC, a natural animal model for the disease, compared to non-AC dogs, suggestive of a perturbation of the canonical Wnt pathway [100].

Up to now, several *in vitro* studies examined the effect of point mutations in the PKP2 gene using iPSC-derived cardiomyocytes (iPS-CMs) from patient affected with AC [101–104]. In all of the cases, mutant iPS-CMs appear unable to reproduce the pathologic phenotype in standard cardiogenic conditions, but they are inclined to lipid accumulation following different treatment with adipogenic stimuli, displaying a functional pro-adipogenic state. Electron microscopy analysis showed larger AC iPS-CMs containing lipid droplets compared with controls, thus suggesting an increased adipogenic potential in these cells. The adipogenic stimuli on the mutant cardiomyocytes seemed to be prevented by GSK3β inhibitor, thus supporting the possible role of the canonical Wnt pathway in AC pathogenesis [103].

Altogether, these findings suggest a central role of the Wnt/β-catenin signaling in the disease pathogenesis and appear to expand the cellular spectrum of AC, commonly recognized as a disease of cardiac myocytes, towards nonmyocyte cells in the heart. However, the direct causal relationship between mutant junctional proteins and perturbed Wnt pathway remains poorly understood.

### Development of Wnt activators as therapeutics for AC

The intricate involvement of Wnt signaling in so many biological processes and disease conditions made this pathway an obvious but yet difficult target for therapeutic intervention. Numerous fields of study have investigated both natural and synthetic compounds able to induce or inhibit Wnt at multiple stages within the pathway. However, despite the evidence of successful methods to rapidly screen thousands of compounds to identify Wnt pathway modulators, unfortunately these molecules cannot be translated yet into the clinical practice [105].

To discover potential chemical modifiers in AC, a zebrafish model with cardiac-specific expression of a truncated form of plakoglobin and manifesting a fully penetrant cardiomyopathy, has been generated and used to screen a library of bioactive compounds for disease modifiers [96] (Table 2). SB216763 molecule has been identified as a compound able to prevent heart failure and to reduce mortality in the fish model. It also rapidly reverses the highly abnormal action potential and corrected the marked decreases in I_Na_ and I_K1_ exhibited by mutant fish myocytes. Interestingly, SB216763 is annotated as an inhibitor of GSK3β thereby increasing canonical Wnt signaling [106]. Additional experiments performed in neonatal rat ventricular myocytes expressing the same mutant plakoglobin and in iPS-CMs from two AC probands with PKP2 mutations supported the SB216763 efficacy in the reversion or prevention of AC pathobiological features. In particular, the distribution of connexin-43 appears to be restored to normal in truncated plakoglobin–expressing cells exposed to SB216763, consistent with previous studies showing the connexin-43 up-regulation by activation of the canonical Wnt pathway [96].

More recently, the SB216763 molecule has been reported to prevent myocyte injury and cardiac dysfunction *in vivo* in two murine models of AC at baseline and in response to exercise (Table 2). GSK3β inhibition improves left ventricle function and survival in sedentary and exercised mice and normalizes ID protein distribution in mutant mouse cardiomyocytes. Redistribution of GSK3β to cardiac IDs appeared to be specific for AC, as it was not evident in myocardia from patients with dilated, hypertrophic or ischemic cardiomyopathy, cardiac sarcoidosis, or giant cell myocarditis. Cytoplasmic distribution of GSK3β was restored in all *in vivo* and *in vitro* models treated with SB216763 Collectively, these data suggest a key role for aberrant GSK3β signaling in the final common disease pathway in AC [97]. However, how localization and activity of GSK3β relate to each other and how the pharmacologic inhibition of this kinase could affect its localization remain to be clarified.

The identification of a molecular activator of the canonical Wnt signaling as a promising pharmacological agent for AC open the door for new mechanism-based therapeutic strategies for patients affected with this disease. Such advances would provide a welcome alternative to current reliance on traditional therapeutic tools (life-style modification, devices, anti-arrhythmic drugs, endocardial and epicardial catheter ablation, implantable cardioverter defibrillator and transplantation) targeting life-threatening ventricular arrhythmias and congestive heart once they occur.

### MicroRNAs: potential linkers between Wnt/β-catenin signaling and AC?

Tissue development, physiology, and functions are strictly regulated by multiple factors. Among them, microRNAs (miRNAs) have been recently described as 22–25 nucleotides long noncoding RNAs which regulate gene expression at post-transcriptional level. This is achieved through imperfect base-pairing with complementary sequences in the 3′UTR of their target mRNA, leading to translational repression or transcript degradation [107]. A single miRNA can regulate multiple genes and, in Human, among the 1,800 detected miRNAs, 800 have been found expressed in the heart [108].

Increasing evidence indicates that miRNAs participate in signaling networks, such as the canonical Wnt/β-catenin signaling, and in promoting or inhibiting the progression of many human diseases, including cardiomyopathies. In the last years, several studies described the correlation between miRNA expression level and the regulation of the Wnt/β-catenin signaling, mostly during cardiac differentiation. Overexpression of miR-499 in rat bone marrow-derived mesenchymal stem cells is sufficient to induce cardiac differentiation, as indicated by increased expression of cardiac specific markers Nkx2.5, GATA4, MEF2C, and cTnl, through Wnt/β-catenin signaling pathway [109]. In particular, miR-499 drives the activation of β-catenin, as shown by the decreased ratio of phosphorylated/dephosphorylated (Ser33/37/Thr41) β-catenin, but the detailed mechanisms involving the Wnt pathway haven't been identified yet [109].

Among the most expressed miRNAs in the heart, miR-1 is known to promote cardiomyocyte differentiation. Lu and colleagues showed that this miRNA suppresses the canonical Wnt/β-catenin signaling in human iPSCs by targeting the receptor Fzd7 and antagonizing the ligand Wnt3A. As a consequence, cardiomyocyte differentiation is promoted and endothelial cell commitment from multipotent cardiovascular progenitors is suppressed [110].

Moreover, several papers reported miR-19b as a negative regulator of the Wnt/β-catenin signaling. Overexpression of miR-19b has been shown to cause aberrant heart tube and chamber development and to regulate the contractile function of the heart in zebrafish embryos by inhibiting the Wnt signaling pathway directly targeting Ctnnb1β [111]. Furthermore, inhibition of GSK3β leads to β-catenin accumulation, together with a partial rescue of the cardiac phenotype [111]. In multipotent murine P19 cells, miR-19b overexpression is responsible for cellular proliferation and differentiation into myocardial cells, and apoptosis inhibition with the parallel decrease of Wnt/β-catenin signaling activation [112]. In this study, luciferase assays show the direct link between the overexpression of miR-19b and the reduction of the expression of WNT1 [112]. Accordingly, miR-19b knock-down results in the inhibition of proliferation and apoptosis of C19 cells, together with the increase of Wnt1 protein levels/β-catenin signaling expression levels [113].

Less frequently, miRNAs have been described to modulate Wnt/β-catenin signaling also during cardiac diseases. Gain and loss of function studies show that miR-145 overexpression leads to an increase in Wnt/β-catenin signaling activity and the downregulation of Dab2, a TGFβ receptor adaptor protein required for cardiac protein expression in mesenchymal stem cells, in response to TGFβ1 [114]. Interestingly, miR-145 is found downregulated in acute myocardial infarction, in association with an increase in Dab2 expression level in cardiomyocytes in the infarct border zone. These results suggest that the Wnt/β-catenin signaling is downregulated in cardiomyocytes after acute myocardial infarction, but further studies are required to clarify the underlying associated mechanism [114].

Interestingly, the expression profile of miRNAs recently performed by qRT-PCR in 24 heart samples from AC patients revealed that miR-21-5p and miR-135b, which are correlated with Wnt and Hippo pathway in cancer, are significantly associated with both the myocardium adiposis and fibrosis, and might to be useful as therapeutic targets [115]. On the other hand, the pathogenic downregulation of miR-184 has been identified in PKP2-deficient cell lines and mouse models of AC [116]. Overexpression of miR-184 had no discernible effects on the transcript levels of selected canonical Wnt signaling targets such as CTNNB1 and TCF7L2 in HL-1 cells and, *vice versa*, the activation of the canonical Wnt signaling does not affect miR-184 levels in the *in vitro* model. The authors show the relationship between the downregulation of miR-184 and perturbations in E2F1 pathway, suggesting a novel mechanism concurring to AC development [116].

Taken together, all these data underline the crucial function of miRNAs in the regulation of theWnt/β-catenin signaling and are suggestive of a potential role of these small RNAs in the pathogenesis and the phenotypic variability of cardiac diseases such as AC. Further efforts will be needed to find out whether miRNAs represent the link between the perturbations at the IDs and the alteration of the Wnt/β-catenin signaling activity observed in AC.

### Concluding remarks

Increasing evidence from experimental studies defines the canonical Wnt/β-catenin signaling as a critical player in the regulation of cardiac function and dysfunctions. To date, the function of this pathway in cardiac hypertrophy, fibrosis and arrhythmias have been well established, however further investigations are required to unravel its role in other pathological conditions. Controversial results have been reported about the involvement of Wnt/β-catenin pathway in the development of AC, an inherited cardiomyopathy determined by mutations mostly in genes encoding for ID proteins and typically characterised by the fibrofatty replacement of the myocardium. Current investigations in zebrafish and mammalian models of AC mainly indicate that suppression of the canonical Wnt/β-catenin signaling promotes cardiac remodelling in affected hearts. The dual function of β-catenin and plakoglobin both in cadherin-mediated adhesion and in gene transcription determines a highly regulated and complex protein network seemingly able to integrate disease signals from the membrane with cellular programs of target gene transcription. However, there are still some questions to be answered, particularly, which signal molecule(s) can play a dominant role in Wnt/β-catenin downregulation upon molecular destabilization of ID junctional structures. A more comprehensive understanding of the dynamics of this signaling in AC cardiomyocytes is therefore required, also for the design of safe and effective drug molecules. Recently, a small molecule activating the Wnt pathway has been experimented and it appears to be remarkably effective in preventing or reversing selected features of the molecular AC pathology. Based on these promising results, Wnt signaling components could represent interesting therapeutic targets for novel treatment strategy in AC, hopefully in the near future. Furthermore, a deeper investigation on the cross-talk between Wnt and other cellular pathways, such as Hippo or TGFβ, may explain other molecular mechanisms leading to AC and assist the design of efficient combinatorial therapeutic protocols. In addition, the relationship between miRNAs-mediated regulation of Wnt signaling, and *vice versa*, needs to be further investigated to contribute in the understanding not only of AC pathogenesis, but also of other, more common forms of heart disease, as well as to provide the basis to develop mechanism-based therapies to treat or prevent diseases at high risk of sudden death.
